# A Clinical Evaluation of Minimally Invasive Ponto Surgery With a Modified Drill System for Inserting Bone-Anchored Hearing Implants

**DOI:** 10.1097/MAO.0000000000003195

**Published:** 2021-05-28

**Authors:** Coosje Jacoba Isabella Caspers, Ivo Joachim Kruyt, Emmanuel Antonius Maria Mylanus, Myrthe Karianne Sophie Hol

**Affiliations:** ∗ Department of Otorhinolaryngology, Donders Center for Neurosciences, Radboud University Medical Center, Nijmegen, The Netherlands; † Department of Otorhinolaryngology/Head and Neck Surgery, University Medical Center Groningen, University of Groningen, Groningen, Netherlands; ‡ Research School of Behavioral and Cognitive Neurosciences, Graduate School of Medical Sciences, University of Groningen, Groningen, Netherlands

**Keywords:** Baha, Bahi, bcd, Drills, Hearing loss, Linear incision technique, Minimally invasive Ponto surgery, Soft tissue preservation, Surgical technique

## Abstract

To compare 6-months outcomes of the modified minimally invasive Ponto surgery (m-MIPS) to both the linear incision technique with soft tissue preservation (LIT-TP), and original MIPS (o-MIPS) for inserting bone-anchored hearing implants (BAHIs).

**Study design:**

Exploratory pilot study with one test group and two historical control groups.

**Setting:**

Tertiary referral center.

**Patients:**

In the test group, 24 patients (25 implants) were prospectively included. Each control group comprised 25 patients (25 implants) who participated in previously conducted clinical trials.

**Interventions:**

The test group received a BAHI using m-MIPS. The two control groups underwent surgery using the LIT-TP and o-MIPS, respectively.

**Main outcome measures:**

Implant survival, implant stability, and surgery-related variables were compared between the test and control groups. Soft tissue status, skin sensibility, and subjective numbness were compared between m-MIPS and LIT-TP only.

**Results:**

Implant survival was comparable between m-MIPS and LIT-TP, whereas implant stability measurements were slightly lower for m-MIPS. M-MIPS resulted in comparable adverse skin reactions and skin sensibility, significantly reduced surgical time and slightly improved subjective numbness, compared with LIT-TP. Between m-MIPS and o-MIPS, no statistically significant differences in implant survival, implant stability and surgical time were observed.

**Conclusions:**

A trend toward lower implant loss rates after m-MIPS was observed, when compared with o-MIPS. M-MIPS seems to be a good alternative to LIT-TP for inserting BAHIs, since most clinical outcomes were either comparable or slightly better for m-MIPS. Upon deciding on which technique to use, larger studies on implant survival should be performed. Furthermore, other aspects such as costs, training aspects and surgical experience should be evaluated.

## INTRODUCTION

Because of its favorable postoperative outcomes, the linear incision technique with soft tissue preservation (LIT-TP) is currently considered the gold standard procedure to insert bone-anchored hearing implants (BAHIs) (1–4). To further reduce postoperative complications, a standardized punch-only procedure called minimally invasive Ponto surgery (MIPS) was developed in 2014 (5,6). Several institutions have already adopted this procedure notwithstanding the high variability in implant loss rates reported, ranging between 0% and 3.9% (6–9) and 12% and 35% (10–12). The high implant loss rates in some studies raised concerns, especially since a nonsignificantly higher implant loss rate was found for MIPS when compared with the LIT-TP and bus-stop technique, respectively (10,12). In line with this, a comparative study of MIPS and LIT-TP conducted at our institution, resulted in a statistically non-significant though higher implant loss rate of 12% for MIPS (13).

Several factors contributing to the high implant loss rates after MIPS have been proposed: 1) the presence of interposed periosteum, 2) incorrect angulation of the drill and/or implant, and 3) inadequate bone cooling resulting in thermal bone necrosis and thus impaired osseointegration (10,12–14). The MIPS drills used in this study are included in an updated MIPS procedure pack available since November 2018 which is currently utilized in several institutions. The design and shape of the drill bits were modified to further improve drill efficiency and osteotomy preparation (15). Additionally, 3-step drilling, as described in the surgical manual, was used instead of 2-step drilling in an attempt to reduce heat generation. To the best of our knowledge, clinical outcomes of the modified MIPS drills (m-MIPS) have not yet been published. Since the modified drills are already in clinical use, we believe it is of importance to investigate the outcomes of m-MIPS, before determining whether this procedure should be considered an equivalent alternative to LIT-TP. We have conducted an exploratory pilot study on clinical outcomes after m-MIPS, focusing on implant survival and stability. Outcomes were compared between m-MIPS and LIT-TP (1), as well as between m-MIPS and MIPS with the original drill design (o-MIPS) (13).

## MATERIALS AND METHODS

### Ethical Considerations

This study was conducted with approval of the local ethical committee and performed according to the guidelines for Good Clinical Practice, ISO14155:2011, and the ethical principles stated by the Declaration of Helsinki (16). All included patients provided written informed consent.

### Study Population

This study consisted of one test group and two control groups. Patients in the test group were prospectively included and underwent BAHI surgery using m-MIPS. Patients in the control groups were already implanted with a BAHI in two previously conducted prospective clinical trials, whereby LIT-TP had been performed in control group 1 (1,2) and o-MIPS in control group 2 (13). Study design, in- and exclusion criteria, outcome measures and follow-up visits were identical among the three groups (1,13). External monitoring was conducted in all studies.

### Surgical Techniques and Follow-Up

In all patients, the Wide Ponto implant® (diameter 4.5 mm, length 4.0 mm, Oticon Medical AB, Askim, Sweden) with abutment (6, 9, or 12 mm) was inserted in a single-stage surgery. All surgeries were performed by experienced ENT-surgeons (EM and MH). Abutment length was chosen based on skin thickness at the implant site, as measured prior to local infiltration.

**FIG. 1:**
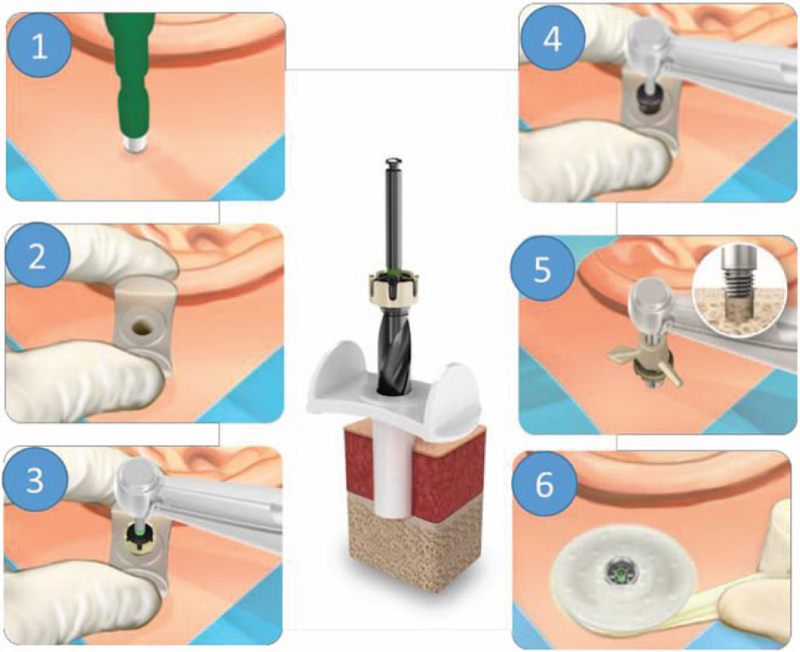
Modified minimally invasive Ponto surgery^∗^: *A*, circular incision is created using a 5-mm biopsy punch (1). The cannula is inserted (2). Three-step drilling is performed (3, 4). The cannula is removed and the implant is inserted (5). A healing cap with dressing is attached to the abutment (6).^*∗*^*Published with permission from Oticon Medical AB*.

In the test group, m-MIPS was conducted (Fig. 1). With this technique, a circular incision is made with a 5-mm biopsy punch, whereafter periosteum is removed with a raspatorium. A cannula is inserted, through which 3-step drilling is performed during continuous irrigation with saline solution and with flushing of the cannula between the drill steps. Three-step drilling consists of the following steps: 1) a guide hole is created using the cannula guide drill with spacer, 2) the hole is deepened for a 4-mm implant using the same drill without spacer, 3) the hole is widened with the cannula widening drill. The cannula is then removed and the implant with premounted abutment is inserted. An insertion indicator is used to check whether full implant insertion is established. If this is not achieved, the implant is manually tightened.

In control group 1, the LIT-TP group, a linear incision has been made down to the periosteum of the skull bone (17). After periosteum removal, a 2-step drilling sequence was performed whereby the guide hole was created to full depth in one step prior to widening of the hole. Following implant-abutment insertion, the skin was closed with sutures and a 5-mm punch hole was created to enable the abutment to penetrate the skin (18). In control group 2, o-MIPS has been performed. This procedure differed from m-MIPS in two aspects: 1) a previous generation drill design was used and 2) 2-step drilling was performed instead of 3-step drilling.

The 2-step drilling sequence, as conducted in the two control groups, represents a deviation from the standard 3-step drilling sequence, recommended by the manufacturer. This 2-step drilling protocol was however performed because, at our tertiary referral center, this procedure is known for its excellent outcomes (2,19).

The aftercare was identical in all groups. Directly after surgery, a healing cap and antibiotic dressing containing hydrocortisone, oxytetracycline and polymyxin-B (Terra-Cortril®) was fixed onto the abutment. Seven days postoperatively, the healing cap was removed and topical application of Terra-Cortril® ointment around the abutment was prescribed twice daily for two weeks. Further follow-up visits were scheduled at 21 days (including sound processor fitting), 12 weeks, and 6 months after surgery.

### Outcome Measures

Main outcome measures were implant survival and stability. Furthermore, surgical time and intraoperative complications were assessed. These outcomes were compared between m-MIPS and LIT-TP, as well as between m-MIPS and o-MIPS. In addition, soft tissue status, skin sensibility around the abutment, subjective numbness and device use were compared between m-MIPS and LIT-TP. Unplanned visits, need for revision surgery and adverse events were recorded.

To assess implant stability, the Implant Stability Quotient (ISQ) was determined by means of resonance frequency analysis directly after complete implant insertion, and at every follow-up visit (1,2,20). For these measurements, the Osstell® ISQ device (Osstell AB, Göteborg, Sweden) and a SmartPeg (type 55) were used. Perpendicular measurements were performed and reported as the highest and lowest scores. Surgical time (in minutes) was defined as punch to complete implant insertion for both o-MIPS and m-MIPS, and as incision to placement of the last suture for LIT-TP. Soft tissue tolerability was assessed by both the Holgers classification (21) and the IPS-scale. The IPS-scale is a new soft tissue assessment scale which includes a standardized treatment advice (22). Because of its recent introduction, the IPS-score was retrospectively assessed in the LIT-TP group. A Holgers ≥2 or IPS score indicating treatment were considered adverse skin reactions. The presence of a skin dehiscence was also reported. For the m-MIPS group, the size of the dehiscence was described in millimeters.

Skin sensibility around the abutment was measured according to the previous trials in which the two control groups participated (1,2,13). Hereby, gnostic and vital sensibility were both tested at six standardized locations around the abutment (1). For this purpose, a broken cotton swab was used; gnostic sensibility was assessed by using the soft end, and vital sensibility was assessed by using the sharp end. The percentage of correct responses was reported and compared between m-MIPS and LIT-TP. Subjective numbness was assessed by means of a Visual Analogue Scale (VAS) ranging from 0 (no numbness) to 10 (complete numbness).

### Statistical Analysis

Achieving the determined sample size for a statically powered study on implant survival was unfortunately not feasible due to the low implant loss rates after BAHI surgery and the small number of patients with an indication for a BAHI. Therefore, we chose to perform an exploratory pilot study with the sample size of the test group set at 25 patients. This sample size was in line with the sample sizes of the control groups (1,2,13). Data analysis was performed using both intention-to-treat (ITT) and per-protocol (PP) populations. For statistical analysis, nonparametric tests were used. Groups were compared using the Fishers nonparametric permutation test for numbness variables, the Mann–Whitney *U*-test for continuous variables, the Mantel Haenzsel chi-square test for ordered categorical variables, the Chi-square test for nonordered categorical variables, and the Fisher's exact test for dichotomous variables. Changes over time were analyzed using the Wilcoxon signed rank test for continuous variables, and the Sign test for dichotomous and ordered categorical variables. The Logrank survival test was used to compare implant survival between groups. In case of premature withdrawal, all collected data to the point of withdrawal were included in the analysis. For the primary variable, missing data were handled using the last-observation-carried-forward method. In case of bilaterally implanted patients, patient characteristics were handled on patient-level, and implant-related characteristics on implant-level. Patients who were included in both test group and control group 2 were treated as two separate subjects in the analysis.

Data analysis were performed by independent external biostatisticians (Statistika Konsultgruppen, Göteborg, Sweden) and conducted according to a predefined statistical plan. According to the predefined plan, no corrections for multiplicity were performed. All statistical tests were two-tailed, conducted at a 0.05 significance level and carried-out using SAS^®^ v9.4 (Cary, NC).

## RESULTS

### Patient Population

In the m-MIPS group, 24 patients (25 implants) were included between September 2018 and June 2019. In the LIT-TP and o-MIPS group, 25 patients (25 implants) were included between February and August 2014, and between June and December 2017, respectively (1,13). Two patients underwent sequential bilateral implantation and were included in both the o-MIPS and m-MIPS group. No baseline differences were found between the test and the two control groups (Table 1). Out of the 74 included patients, five patients did not complete the 6-month follow-up because of implant loss (one in m-MIPS group and three in o-MIPS group) and abutment removal (one in o-MIPS group). Six patients were excluded from the PP population, comprising the five prematurely withdrawn patients, and one bilaterally implanted patient who was 5 weeks late for the 6-month visit after m-MIPS. Below, outcomes of the ITT population are described (see also Table 2). Outcomes of the PP population are presented in supplemental digital content 1.

**TABLE 1:** Patient and surgical characteristics for the test group and two control groups

Variable^*a*^	Modified MIPS *Test Group*	LIT-TP *Control Group 1*	Original MIPS *Control Group 2*
Patient Variables	n = 24	n = 25	n = 25
Gender, n (%)			
Male	9 (38)	15 (60)	9 (36)
Female	15 (63)	10 (40)	16 (64)
Age in years, mean (SD)	53 (14)	52 (13)	60 (13)
Ethnicity, n (%)			
Caucasian	23 (96)	25 (100)	25 (100)
Hispanic	1 (4)	0 (0)	0 (0)
Smoking, n (%)	6 (25)	4 (16)	5 (20)
Relevant diseases, n (%)			
Diabetes Mellitus^*b*^	3 (13)	0 (0)	3 (12)
Skin disease	1 (4)	1 (4)	0 (0)
Chronic steroid use	0 (0)	1 (4)	0 (0)
Indication, n (%)			
Acquired conductive/mixed	17 (71)	21 (84)	20 (80)
Congenital conductive	0 (0)	1 (4)	1 (4)
Single-sided deafness	7 (29)	3 (12)	4 (16)
Bilateral implantation, n (%)	1 (4)	0 (0)	0 (0)

Surgical (implant) variables	n = 25	n = 25	n = 25
Intraoperative complication, n (%)			
Drilling into vein	2 (8)	4 (16)	2 (8)
Dura mater exposed	1 (4)	2 (8)	0 (0)
Difficult implant insertion^*c*^	3 (6)	0 (0)	0 (0)
Abutment length, n (%)			
6 mm	1 (4)	0 (0)	1 (4)
9 mm	14 (56)	17 (68)	14 (56)
12 mm	10 (40)	8 (32)	10 (40)
Surgery time in minutes, mean (SD)	6.2 (2.7)	20.8 (4.3)	6.4 (2.4)

LIT-TP indicates linear incision technique with soft tissue preservation; MIPS, minimally invasive Ponto surgery; SD, standard deviation.

a Variables were compared between modified MIPS and LIT-TP, and between modified MIPS and original MIPS.

b Type 2 diabetes mellitus with stable blood glucose levels and treatment with dietary restrictions and/or oral diabetes medication.

c Implant needed to be repositioned in the existing punch-hole because of incomplete insertion (two cases) or an incorrect angle (one case).

**TABLE 2:** Outcome measures compared between test and control groups

Outcome Measure	m-MIPS *Test*	LIT-TP *Control 1*	*p* *m-MIPS* *vs LIT-TP*	o-MIPS *Control 2*	*p* *m-MIPS vs o-MIPS*
Implant loss 0–6 months, n (%)	n = 25	n = 25		n = 25	
Implant loss	1 (4.0)	0 (0.0)	0.32	3 (12.0)	0.30
Mean AUC ISQ 0–6 months	n = 25	n = 25			
6-mm abutment, ISQ-low^*a*^	69.0			68.3	
6-mm abutment, ISQ-high^*a*^	69.2			70.6	
9-mm abutment, ISQ-low	57.1 (2.8)	59.1 (2.2)	0.065	57.1 (3.3)	0.87
9-mm abutment, ISQ-high	58.6 (2.4)	60.6 (2.4)	0.041	59.5 (3.5)	0.35
12-mm abutment, ISQ-low	48.8 (3.5)	52.8 (3.9)	0.10	48.9 (4.1)	0.34
12-mm abutment, ISQ-high	50.5 (3.1)	54.8 (3.7)	0.037	51.3 (3.9)	0.97
Maximum Holgers 0–6 months, n (%)^*b*^	n = 25	n = 25			
0	18 (72)	11 (44)			
1	5 (20)	7 (28)			
2	2 (8)	4 (16)			
3	0 (0)	3 (12)			
4	0 (0)	0 (0)	0.028		
Maximum I-, P-, S-scores 0–6 months, n (%)^*b*^	n = 25	n = 25			
I-score (inflammation)					
0	5 (21)	12 (48)			
1	17 (71)	7 (28)			
2	2 (8)	2 (8)			
3	0 (0)	4 (16)			
4	0 (0)	0 (0)	0.87		
P-score (pain)					
0	20 (83)	15 (60)			
1	4 (17)	10 (40)			
2	0 (0)	0 (0)	0.11		
S-score (skin height)					
0	23 (96)	17 (68)			
1	1 (4)	8 (32)			
2	0 (0)	0 (0)	0.027		
Sensibility at 6 months, mean % (SD) ^*c*^	n = 25	n = 25			
Total sensibility	100 (0.0)	98.0 (4.4)	0.048		
Gnostic sensibility	100 (0.0)	96.7 (8.3)	0.11		
Vital sensibility	100 (0.0) (0.0)	99.3 (3.3) (3.(3.3)	1.00		
Subjective numbness at 6 months, mean (SD) (SD_ (SD)	n *=* 24	n *=* 25			
VAS	0.21 (1.0)	0.36 (1.1)	0.12		
Sound processor use at 6 months	n *=* 24	n *=* 25			
Daily users, n (%)	23 (96)	19 (76)	0.11		
Reason unplanned visit, n					
Pain at implant side	1	2		0	
Inflammation at implant side	1	4		2	
Implant loss	1	0		3	
Abutment removal	0	0		1	
Postoperative fever	0	1		0	
Implant-related adverse event, *n*					
Pain at implant side	1	1		3	
Recurrent inflammation at implant side	2	0		3	
Bleeding around implant	2	0		0	
Small wound next to abutment	0	0		1	
Persistent itch	1	0		2	
Surgery-related adverse event, n					
Postoperative fever	0	1		0	
Postoperative headache	4	0		2	
Postoperative dizziness	1	0		2	

Results are presented for the ITT population. AUC, area under the curve; ISQ, implant stability quotient; LIT-TP, linear incision technique with soft tissue preservation; MIPS, minimally invasive Ponto surgery; m-MIPS, modified MIPS; o-MIPS, original MIPS; SD, standard deviation; VAS, visual analogue scale.

a Only two 6-mm abutments were used, one in the modified MIPS group and one in the original MIPS group.

b All visits including unplanned visits.

c Last-observation-carried-forward (LOCF) method was used. Without LOCF, mean total, gnostic and vital sensibility were similar, but the *p*-values were slightly different (*p* = 0.057 for total sensibility, *p* = 0.11 for gnostic sensibility and *p* = 1.00 for vital sensibility).

### Surgery

All surgical procedures were performed without major intraoperative complications (Table 1) or conversions to another surgical technique. Compared with LIT-TP, m-MIPS reduced the previously defined “surgical time” with 70% (*p* < 0.0001). Surgical time for m-MIPS and o-MIPS was comparable.

### Unplanned Visits and Adverse Events

In all patients, a total of 16 unplanned visits, 10 surgery-related- and 15 implant-related adverse events were reported (Table 2). All adverse events were considered mild to moderate and resolved either spontaneously or with the use of local antibiotic ointment.

### Implant Survival and Stability

In the m-MIPS group, one implant was lost within 10 weeks postoperatively, preceded by complaints of pain. In the LIT-TP no implant loss occurred, and in the o-MIPS three implants were lost (two spontaneous, one after trauma), all within 10 weeks postoperatively. No significant differences in implant loss were observed between m-MIPS and LIT-TP (*p* = 0.32), nor between m-MIPS and o-MIPS (*p* = 0.30). In both the m-MIPS and o-MIPS group, a change to a shorter abutment was performed in one patient because of complaints related to too much protrusion. Furthermore, one abutment was removed in the o-MIPS group due to the patient being unsatisfied with the device.

**FIG. 2:**
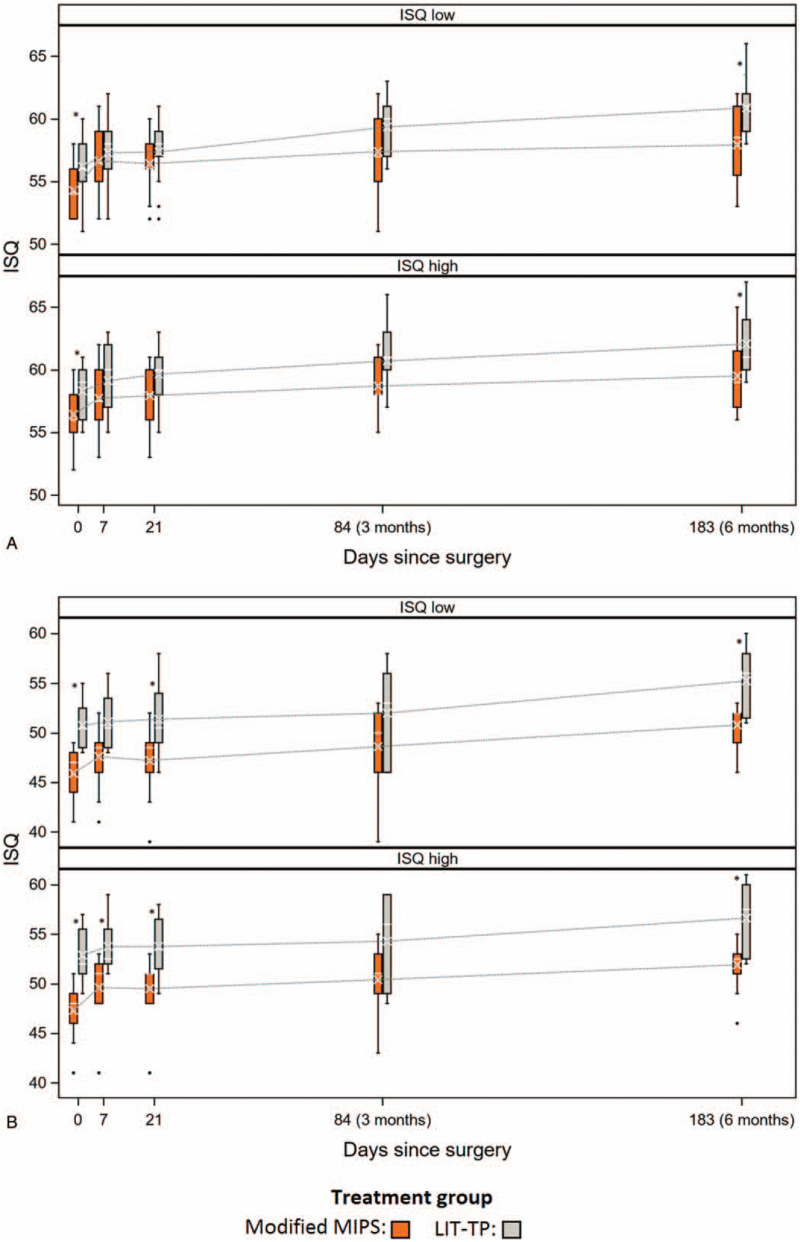
ISQ values for the modified MIPS (in orange) and LIT-TP (in grey) group at baseline, 7 days, 21 days, 3 months, and 12 months after surgery for the 9-mm (Fig. 2A) and the 12-mm abutment (Fig. 2B). Outliers are shown as dots. Significant differences between groups are marked with an asterisk. MIPS indicates minimally invasive Ponto surgery; LIT-TP, linear incision technique with soft tissue preservation.

For the 9-mm and 12-mm abutment, the mean 0-6 month area under the curve (AUC) of the ISQ-high was significantly higher for LIT-TP compared with m-MIPS, whereas the 0-6 month ISQ-low was comparable between groups (Table 2). Similar 0-6 month AUC ISQ values were found for m-MIPS and o-MIPS. For m-MIPS and LIT-TP, ISQ values were either similar or slightly lower for m-MIPS, depending on the time point of assessment (Fig. 2). For m-MIPS and o-MIPS, ISQ-high and -low were comparable across visits. ISQ-high and -low improved significantly over time in all three treatment groups.

### Soft-Tissue Status

**FIG. 3:**
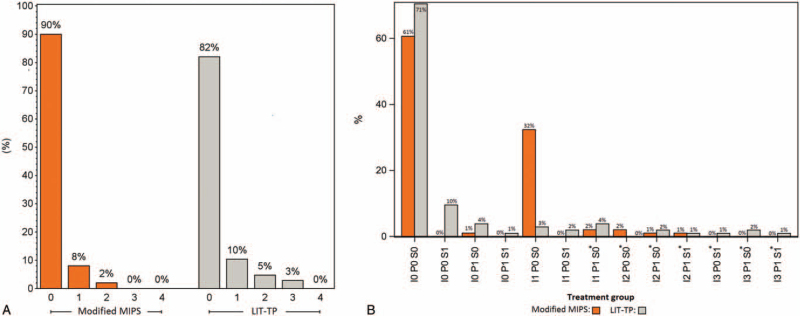
Soft tissue reactions across all visits according to the Holgers scale, *A* and the IPS-scale, *B* for the modified MIPS group (orange) and LIT-TP group (grey). Holgers ≥ 2 and IPS scores indicating treatment were considered to be adverse skin reactions. Adverse IPS scores are marked with an asterisk. MIPS indicates minimally invasive Ponto surgery; LIT-TP, linear incision technique with soft tissue preservation.

A skin dehiscence was observed in 72% of the m-MIPS patients but in none of the LIT-TP patients (*p* < 0.0001). All skin dehiscences consisted of a small gap between the abutment surface and the surrounding skin with a median width of 2 mm (range 0.5–3 mm). The patients with skin dehiscence did not experience any discomfort. At the 12-week visit all skin dehiscences were healed. Within this time period, no adverse Holgers scores were observed in these patients. Holgers and IPS scores across visits are presented in Fig. 3. The Holgers scores differed significantly between groups at 6 months, with worse scores for the LIT-TP group (*p* = 0.049). In line with this, maximum Holgers across visits were higher for LIT-TP (Table 2). Furthermore, albeit not reaching statistical significance, adverse Holgers scores were reported in 8% and 28% of the m-MIPS and LIT-TP group, respectively (*p* = 0.14). A significant difference in the distribution of total IPS-scores was found between m-MIPS and LIT-TP at 7 days, 21 days, and 12 weeks after surgery (*p* < 0.0001 at 7 days; *p* = 0.0007 at 21 days and *p* < 0.0081 at 12 weeks). A statistically higher Inflammation (I)-score was found for m-MIPS at 7 and 21 days, and a statistically higher Skin height (S)-score and I-score for LIT-TP at 12 weeks and 6 months, respectively. The Pain (P)-score did not differ between groups. Across all visits, the maximum I- and P-score did not differ significantly between groups, whereas the maximum S-score was significantly higher for LIT-TP (*p* = 0.027; Table 2). When comparing adverse IPS-scores at the different visits, no statistical difference was found between groups. Across visits, adverse IPS-scores were reported in 17% and 36% of the m-MIPS and LIT-TP group, respectively (*p* = 0.23). All adverse skin reactions were successfully treated with antibiotic ointment. None of the patients required revision surgery.

### Sensibility and Subjective Numbness

**FIG. 4:**
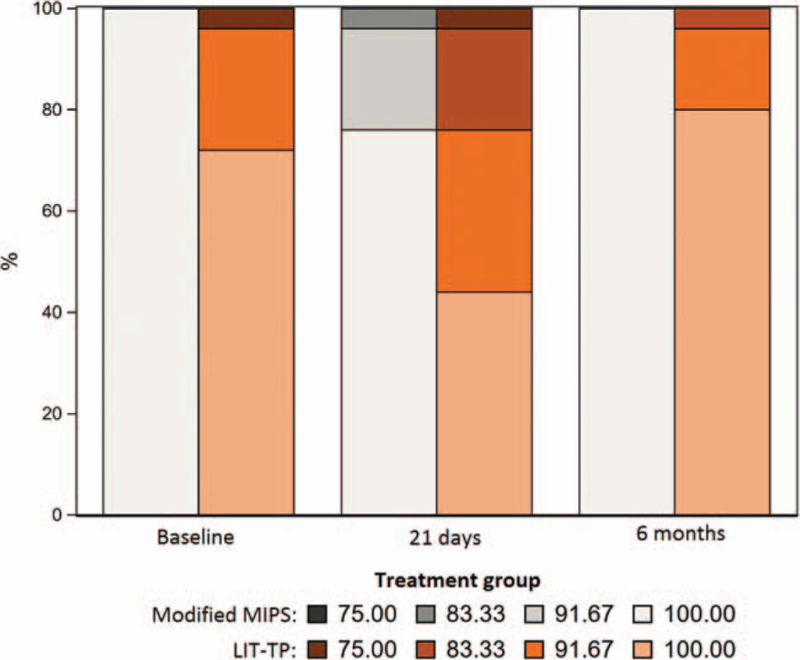
Total skin sensibility around the abutment at baseline, 21 days and 6 months after modified MIPS (presented in grey) and LIT-TP (presented in orange). MIPS indicates minimally invasive Ponto surgery; LIT-TP, linear incision technique with soft tissue preservation.

Total sensibility across visits is presented in Fig. 4. At baseline, mean total sensibility was significantly better for m-MIPS compared with LIT-TP (100% vs. 97%, *p* = 0.009). At 21 days after surgery, sensibility had decreased in both groups with a significantly higher sensibility for m-MIPS (97.7% vs 93%, *p* = 0.017). At 6 months, skin sensibility was also favorable for m-MIPS (Table 2). However, change in sensibility from baseline to 21 days and 6 months did not significantly differ between groups. In general, skin sensibility was back to baseline sensibility at 12 weeks for m-MIPS and at 6 months for LIT-TP, respectively. It must however be noted that skin sensibility was not measured in the LIT-TP group at the 12 week visit. VAS scores for subjective numbness were at baseline levels at 21 days for m-MIPS and at 6 months for LIT-TP. Subjective numbness was significantly better for m-MIPS at 21 days (VAS 0.2 [SD 1.0] vs VAS 2.2 [SD 2.7]; *p* < 0.001), and comparable between groups at 6 months (Table 2).

### Device Use

Device use is presented in Table 2. For the seven patients who did not use their sound processors on a daily base, the median use was 4 days a week (range 0–5).

## DISCUSSION

In terms of intra- and postoperative complications, both MIPS and the linear incision technique with soft tissue preservation (LIT-TP) come across as safe techniques to insert BAHIs. Implant survival after 6-month follow-up was comparable between groups with an implant survival rate of 96% for m-MIPS and 100% for LIT-TP. M-MIPS significantly reduced surgical time with 70%, compared with LIT-TP, whereas adverse skin reactions across visits and device use were comparable between these groups. With regards to skin sensibility, our data suggest that numbness is no longer a postoperative side effect of BAHI surgery when using tissue-preserving techniques including MIPS. When comparing m-MIPS and o-MIPS, no statistically significant differences in implant stability, implant survival, surgical time and intraoperative complications were found.

Highly variable implant loss rates have been reported for o-MIPS in the literature (6–12). Our findings might show a tendency toward lower implant loss rates with m-MIPS compared with o-MIPS, with implant loss rates of 4% versus 12%. It is plausible these findings are attributed to the modified drill design in combination with the use of the 3-step drilling sequence.

### Strengths and Limitations

This is the first study to evaluate clinical outcomes of m-MIPS and to compare these to outcomes of the LIT-TP and o-MIPS. All data was prospectively collected and all patients were scheduled according to an identical follow-up scheme. Additionally, outcomes were measured in a standardized manner. Due to the use of historical control groups, randomization and a blinded follow-up were not possible. Sample size of the test group was determined based on the number of patients in the control groups rather than a statistical power calculation.

### Interpretation of Findings

In a preclinical study comparing the drill set design of m-MIPS and o-MIPS, the modified drills were found to generate significantly less heat, except in cases of impaired irrigation where they performed equally (15). In line with this, a low implant loss rate of 4% was found for m-MIPS in the current study, whereas an implant loss rate of 12% was previously found for o-MIPS. Although a follow-up of 6 months seems relatively short, in previous publications on o-MIPS, all implant losses occurred within 3 months after implantation (10–13). These early implant losses support the hypothesis that implant loss after o-MIPS may be a result of impaired osseointegration caused by overheated bone. A recent in vitro study evaluating drill components used for BAHI surgery demonstrated the dependence of temperature increase during ostomy preparation on multiple factors such as drill design, irrigation and drilling procedure (23) The combination of the modified drill design and the 3-step drilling protocol with adequate irrigation, therefore seems promising with regards to implant survival. In contrast, caution is required when drawing firm conclusions regarding implant loss rates. One could argue, however, based on this exploratory pilot study, a trend toward better implant survival might be expected using m-MIPS compared to o-MIPS. In order to draw firm conclusions, further research on (long-term) implant survival with adequate sample sizes is warranted.

As for ISQ-measurements, both the ISQ-high and -low were significantly lower for m-MIPS compared with LIT-TP at several follow-up visits. Additionally, the mean 0-6 month AUC of the ISQ-high was significantly lower for m-MIPS as well. The meaning and relevance of individual ISQ-values is subject to debate and individual values should therefore not be interpreted (20). The trend in ISQ-values over time in a population is thought to be a more relevant measure of implant stability (20). In all three study groups, both ISQ-low and -high did increase over time.

The current outcomes on skin sensibility and soft tissue status after m-MIPS, in comparison with LIT-TP, are in line with previously conducted studies comparing o-MIPS and LIT-TP, with better subjective numbness for MIPS and comparable skin sensibility and adverse skin reactions between groups (12,13). Significantly lower maximum Holgers scores and a trend toward fewer adverse Holgers scores were observed for m-MIPS compared with LIT-TP. A reduction in adverse skin reactions with m-MIPS seems reasonable, since tissue damage is minimal and the vascularity surrounding the implant is left intact. The tendency toward fewer adverse Holgers scores has been observed in studies evaluating o-MIPS as well, but no significant differences have been found when compared with LIT-TP (12,13). In this study, the IPS scale was also used (22). Whereas the Holgers scale was designed to determine soft tissue status at 3 months after implantation, the IPS scale was developed to assess inflammation, as well as skin height and the presence of pain at any moment after surgery. These differences are reflected by the number of adverse skin reactions according to each scale: adverse IPS scores were reported more frequently than adverse Holgers scores. The I-score, which includes the parameter skin integrity, was significantly higher for m-MIPS at 7 and 21 days after surgery, due to the high incidence of skin dehiscences. The higher S-scores at 12 weeks for LIT-TP all concerned S1 scores, indicating that revision surgery was not required. The higher I-scores for LIT-TP at 6 months corresponded with higher Holgers scores in this group, suggesting the presence of erythema, edema or granulation tissue in these patients.

### Other Considerations

When deciding whether BAHIs should be inserted with m-MIPS or LIT-TP, we believe it is important to take aspects like costs, training, surgical experience and perhaps also patients’ preferences, into account. Surgical time is shorter for m-MIPS compared with LIT-TP and a reduction of costs might therefore be expected. In contrast, the total time spent in the operation theater and equipment-related costs should also be taken into consideration. When training surgical residents, or when performing BAHI surgery in (especially young) patients with thin cranial bones or cranial malformations, m-MIPS may be a less appropriate technique. Furthermore, when performing m-MIPS, experience with LIT-TP is of importance since conversion to an open technique could be required, for example in case of a major bleeding (7).

## CONCLUSION

In this exploratory pilot study, MIPS and the linear incision technique with soft tissue preservation both resulted in favorable clinical outcomes with low intra- and postoperative complication rates and are considered safe techniques to insert BAHIs. A tendency toward lower implant loss rates was observed when using the second generation of MIPS (m-MIPS), when compared to the first generation. Therefore, m-MIPS seems be a good alternative to LIT-TP. However, upon deciding on which technique to use, data of long-term studies with adequate sample sizes and cost-benefit studies are necessary.

## Supplementary Material

Supplemental Digital Content
