# Supplementary material for: A Clinical Evaluation of Minimally Invasive Ponto Surgery With a Modified Drill System for Inserting Bone-Anchored Hearing Implants
Source: Otol Neurotol. 2021 May 28;42(8):1192–200. doi: 10.1097/MAO.0000000000003195 (PMC8867490; doi:10.1097/MAO.0000000000003195)
Supplement: Supplemental Digital Content [file mao-42-1192-s001.docx]

**Supplemental digital content 1.** Outcome measures compared between test and control groups. Results are presented for the PP population. MIPS indicates minimally invasive Ponto surgery; m-MIPS, modified MIPS; o-MIPS, original MIPS; LIT-TP, linear incision technique with soft tissue preservation; SD, standard deviation; AUC, area under the curve; ISQ implant stability quotient; VAS, visual analogue scale.

| **outcome measure** | **m-MIPS**  *Test* | **LIT-TP**  *Control 1* | **p-value** *m-MIPS*  *vs LIT-TP* | **o-MIPS**  *Control 2* | **p-value**  *m-MIPS vs o-MIPS* |
| --- | --- | --- | --- | --- | --- |
| Mean AUC 0-6 months | *n=22* | *n=25* |  | *n=21* |  |
| 6-mm abutment –ISQ low ^a^ | 69.0 |  |  | 68.3 |  |
| 6-mm abutment – ISQ high ^a^ | 69.2 |  |  | 70.6 |  |
| 9-mm abutment – ISQ low | 57.8 (2.7) | 59.1 (2.2) (2.2)(2.2(2.3) | 0.28 | 58.0 (2.6) | 0.78 |
| 9-mm abutment – ISQ high | 58.9 (2.5) | 60.6 (2.4) | 0.14 | 60.2 (3.1) | 0.17 |
| 12-mm abutment – ISQ low | 48.8 (3.5) | 52.8 (3.9) | 0.10 | 49.6 (4.0) | 0.51 |
| 12-mm abutment – ISQ high | 50.5 (3.1) | 54.8 (3.7) | 0.037 | 51.7 (4.1) | 0.89 |
| Maximum Holgers 0-6 months, n (%) ^b^ | *n=22* | *n=25* |  |  |  |
| 0 | 17 (77) | 11 (44) |  |  |  |
| 1 | 4 (18) | 7 (28) |  |  |  |
| 2 | 1 (5) | 4 (16) |  |  |  |
| 3 | 0 (0) | 3 (12) |  |  |  |
| 4 | 0 (0) | 0 (0) | 0.009 |  |  |
| Maximum I-, P-, S-scores 0-6 months, n (%) ^bbbbbba^ | *n=22* | *n=25* |  |  |  |
| I-score (Inflammation) |  |  |  |  |  |
| 0 | 5 (23) | 12 (48) |  |  |  |
| 1 | 16 (73) | 7 (28) |  |  |  |
| 2 | 1 (5) | 2 (8) |  |  |  |
| 3 | 0 (0) | 4 (16) |  |  |  |
| 4 | 0 (0) | 0 (0) | 0.74 |  |  |
| P-score (Pain) |  |  |  |  |  |
| 0 | 20 (91) | 15 (60) |  |  |  |
| 1 | 2 (9) | 10 (40) |  |  |  |
| 2 | 0 (0) | 0 (0) | 0.02 |  |  |
| S-score (Skin height) |  |  |  |  |  |
| 0 | 22 (100) | 17 (68) |  |  |  |
| 1 | 0 (0) | 8 (32) |  |  |  |
| 2 | 0 (0) | 0 (0) | 0.007 |  |  |
| Sensibility at 6 months, mean % (SD) | *n=22 N=25* | *n=25* |  |  |  |
| Total sensibility | 100 (0.0) | 98.0 (4.4) | 0.07 |  |  |
| Gnostic sensibility | 100 (0.0) | 96.7 (8.3) | 0.14 |  |  |
| Vital sensibility | 100 (0.0) (0.0) | 99.3 (3.3) (3.(3.3) | 1.00 |  |  |
| Subjective numbness at 6 months, mean (SD) (SD_ (SD) | *n=22* | *n=25* |  |  |  |
| VAS | 0.23 (1.1) 0.0 (0.0) | 0.36 (1.1) | 0.15 |  |  |
| Sound processor use at 6 months | *n=22* | *n=25* |  |  |  |
| Daily users, n (%) | 21 (96) | 19 (76) | 0.11 |  |  |

^a^ Only two 6-mm abutments were used, one in the modified MIPS group and one in the original MIPS group.

^b^ All visits including unplanned visits.
